# LSPR Coupling and Distribution of Interparticle Distances between Nanoparticles in Hydrogel on Optical Fiber End Face

**DOI:** 10.3390/s17122723

**Published:** 2017-11-25

**Authors:** Harald Ian Muri, Dag Roar Hjelme

**Affiliations:** Department of Electronic Systems, Norwegian University of Science and Technology, Gunnerus gate 1, 7012 Trondheim, Norway; dag.hjelme@ntnu.no

**Keywords:** reflection-based FO systems, smart hydrogel, LSPR coupling, nanoplasmonics, point dipole model, Mie scattering, proof-of-concept, interparticle distance distribution, pH sensor

## Abstract

We report on a new localized surface plasmon resonance (LSPR)-based optical fiber (OF) architecture with a potential in sensor applications. The LSPR-OF system is fabricated by immobilizing gold nanoparticles (GNPs) in a hydrogel droplet polymerized on the fiber end face. This design has several advantages over earlier designs. It dramatically increase the number nanoparticles (NP) available for sensing, it offers precise control over the NP density, and the NPs are positioned in a true 3D aqueous environment. The OF-hydrogel design is also compatible with low-cost manufacturing. The LSPR-OF platform can measure volumetric changes in a stimuli-responsive hydrogel or measure binding to receptors on the NP surface. It can also be used as a two-parameter sensor by utilizing both effects. We present results from proof-of-concept experiments exploring the properties of LSPR and interparticle distances of the GNP-hydrogel OF design by characterizing the distribution of distances between NPs in the hydrogel, the refractive index of the hydrogel and the LSPR attributes of peak position, amplitude and linewidth for hydrogel deswelling controlled with pH solutions.

## 1. Introduction

Fiber optic (FO) sensors based on local surface plasmon resonance (LSPR) have been proposed in various configurations over the last decade [1,2,3,4]. The most important features of LSPR FO sensors are label-free sensing, fast response time, high sensitivity, high selectivity, simplified optical design and remote sensing capabilities. The label-free sensing can also be multi-parametric by spectrally resolving different LSPR observed for noble metal nanostructures (NMNS) of different sizes and shapes [5]. LSPR-based FO sensors usually utilize NMNS interacting with the evanescent field at the optical fiber (OF) core-cladding interface or with the light at the OF end face [2,4]. The use of the OF end face offers simpler manufacturing methods compared to utilizing the evanescent field since there is no need for cladding removal. The techniques available for immobilization of NMNS on an OF end face are limited to essentially a monolayer manufactured by photolithographic structuring of metal film, thermal nucleation of metal film or random immobilization of nanoparticles (NP).

**Figure 1:**
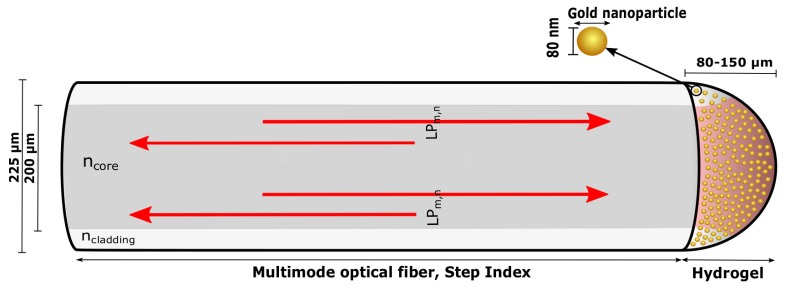
Illustration of the multimode (MM) OF with hydrogel containing gold nanoparticles (GNPs) immobilized on a fiber end face. Visible light is guided in the fiber core with the numerical aperture colored with red in the hydrogel.

This paper reports on the development and the characterization of a new LSPR-based OF architecture in reflection prepared by immobilizing gold nanoparticles (GNP) in a hemispherical hydrogel on the OF end face with potential to be used in sensor applications, as shown in Figure 1.

The LSPR-based OF system is configured using a multimode (MM) OF with a semi-spherical hydrogel containing spherical GNPs immobilized on OF end face. This configuration ensures a strong LSPR signal as a result of the high numerical aperture (NA) exciting a large fraction of the GNPs and effective collection of scattering from the LSPR of the GNPs. The advantages over earlier designs lies in the increase of the number of GNPs available for sensing, the precise control over the GNP density, having free standing single GNPs distributed in a three-dimensional aqueous environment, and low cost manufacturing. The LSPR OF system can be utilized to detect volumetric changes in the hydrogel, receptor-analyte recombination on the GNP surface or chemical concentrations with surface-enhanced Raman scattering (SERS). By using NPs of different sizes, shapes or material compositions, they can also be used for multianalyte sensing in complex mixtures.

We present proof-of-concept experiments where we explore the LSPR and interparticle distance distribution attributes of the GNP-hydrogel OF design. The LSPR properties and interparticle distances of GNP-hydrogel are assessed for the increasing number density (ND) of GNPs, as well as for the increasing polymer density of the hydrogel. The polymer density is controlled by inducing swelling or deswelling with pH solutions. The ND is controlled by changing the initial ND of GNPs in the hydrogel, as well as by inducing swelling and deswelling of the hydrogel. Increasing ND reduces the distance between the neighboring GNPs that may induce electrostatic interaction between the particles, causing a shift in the LSPR. Increasing polymer density may also result in an LSPR shift due to increasing refractive index (RI). The nearest-neighbor distribution function (NNDF) is therefore computed for increasing ND of GNPs embedded in the hydrogel to assess the distribution of interparticle distances, whereas the RI as a function of hydrogel deswelling is investigated based on the estimation of the mole fraction of polymer and water. The LSPR peak position, amplitude and full width at half maximum (FWHM) of the reflectance from GNP-hydrogel are characterized for the hydrogel deswelling stimulated with pH solutions and for the increase in ${\mathrm{ND}}_{0}$. In addition, the sources of reflection are investigated with respect to the LSPR reflectance spectrum to determine possible errors included in the LSPR peak position value. Since the LSPR peak position is measured for the hydrogel swelling degree induced by different acidic solutions, the LSPR FO design is also represented as a pH sensor.

## 2. LSPR Attributes of GNPs Immobilized in a Hydrogel

### 2.1. Scattering of GNP-Hydrogel

**Figure 2:**
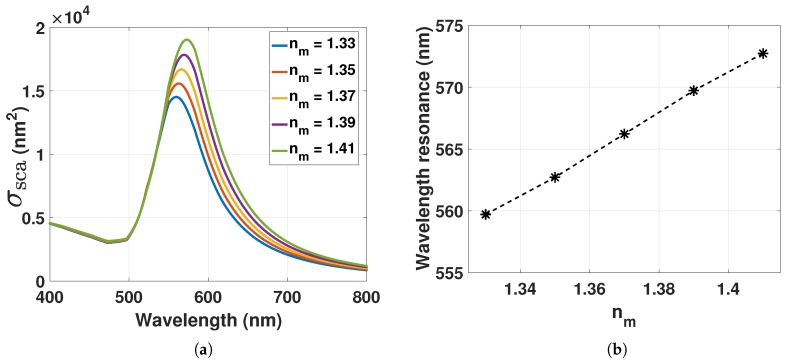
(**a**) Scattering cross-section computed from Equation (1) with L=1 of GNP with 80 nm in diameter for increasing refractive index (RI) of the surrounding medium; (**b**) LSPR peak position from Figure 2a as a function of increasing RI.

The absorption and scattering of incident light on NMNS depends on the optical frequency, particle size and shape, the dielectric environment and the optical constants of the metal. The reflection from GNP embedded in hydrogel immobilized on OF end face is a result of both scattering and absorption. However, in our case with 80-nm GNPs, the reflection will be dominated by the scattering. With sufficient low GNP density and the absence of dipole-dipole interactions, the scattering cross-section of GNPs embedded in hydrogel can be described by Mie theory for spherical particles [6],

(1) $$\sigma_{\mathrm{sca}}=\frac{P_{\mathrm{sca}}}{I_{\mathrm{inc}}}=\frac{2\pi }{{\left|k\right|}^{2}}\sum_{L=1}^{\infty }\left(2L+1\right)(|a_{L}{|}^{2}+|b_{L}{|}^{2})$$

where $P_{\mathrm{sca}}$ is the scattered power, $I_{\mathrm{inc}}$ is the incident plane wave intensity, *L* are integers representing a dipole for L=1 or multipoles for L>1 and *k* is the incoming wavevector. $a_{L}$ and $b_{L}$ are parameters composed of the Riccati–Bessel functions $\psi_{L}$ and $\chi_{L}$,

(2a) $$a_{L}=\frac{m\psi_{L}\left(mx\right)\psi_{L}^{\prime }\left(x\right)-\psi_{L}^{\prime }\left(mx\right)\psi_{L}\left(x\right)}{m\psi_{L}\left(mx\right)\chi_{L}^{\prime }\left(x\right)-\psi_{L}^{\prime }\left(mx\right)\chi_{L}\left(x\right)}$$

(2b) $$b_{L}=\frac{\psi_{L}\left(mx\right)\psi_{L}^{\prime }\left(x\right)-m\psi_{L}^{\prime }\left(mx\right)\psi_{L}\left(x\right)}{\psi_{L}\left(mx\right)\chi_{L}^{\prime }\left(x\right)-m\psi_{L}^{\prime }\left(mx\right)\chi_{L}\left(x\right)}$$

where the primes represents the first differentiation with respect to the argument in the parenthesis, $x=k_{m}r$, *r* is the radius of the particle, $k_{m}$ is the wavenumber of the incident light within a medium, $m=\frac{\tilde{n}}{n_{m}}$, $n_{m}$ is the real refractive index of the surroundings of the metal and $\tilde{n}=n_{R}+in_{I}$ is the complex refractive index of the metal. For a dipole with x<<1, one can use an approximation of the Riccati–Bessel functions given by Bohren and Huffman to express the scattering cross-section as [7],

(3) $$\sigma_{\mathrm{sca}}=\frac{32\pi^{4}{\varepsilon_{m}}^{2}V^{2}}{\lambda^{4}}\left[\frac{{\left(\varepsilon_{1}-\varepsilon_{m}\right)}^{2}+{\left(\varepsilon_{2}\right)}^{2}}{{\left(\varepsilon_{1}+2\varepsilon_{m}\right)}^{2}+{\left(\varepsilon_{2}\right)}^{2}}\right]$$

where $\varepsilon_{1}$ and $\varepsilon_{2}$ are the real and imaginary components, respectively, of the complex metal dielectric function $\tilde{\varepsilon }\left(\lambda \right)=\varepsilon_{1}+i\varepsilon_{2}$, $\varepsilon_{m}$ is the dielectric constant of the surrounding medium, *V* is the particle volume, and λ is the wavelength of the incident light. Maximum scattering in Equation (3) occurs when the condition of Reε˜(λ)=−2εm is met. If the dielectric medium around the GNP changes, the wavelength of the LSPR changes. The scattering of 80-nm GNPs can be computed by Equation (1) with L=1 as shown in Figure 2 for different RI of the surrounding medium ($n_{m}$).

Optical constants of bulk gold where taken from Johnson and Christy [8]. The redshift for increasing RI will also be associated with a spectral broadening and an increasing amplitude. By using the Drude model, the LSPR peak position as a function of $n_{m}=\sqrt{\varepsilon_{m}}$ within a sufficiently narrow range can be described as,

(4) $$\lambda_{\max}=\lambda_{p}\sqrt{2{n_{m}}^{2}+1}$$

where $\lambda_{p}$ is the plasma oscillation wavelength of the bulk metal [9]. A deswelling hydrogel increases the polymer density, which also increases the probability of having polymer chains in close proximity to the GNPs. The polymer chains in close proximity to the plasmonic wave of the GNPs increase the local RI, which will redshift the LSPR.

### 2.2. Electrostatic Interactions between Dipoles of GNPs in Hydrogel

Considering a sufficiently high density of GNPs immobilized in the hydrogel, a dipole-dipole interaction between them will occur due to the short interparticle distances. For center-to-center interparticle distances *d* less than 5r, the resonance condition Reε˜(λ)=−2εm found from Equation (3) should be corrected by including a dependence on the ND [10]. For two spheres of equal size in close proximity to each other with near-field coupling in the longitudinal mode, the dispersion equation can be described by the point dipole model [11,12],

(5) $$1-4\frac{\alpha_{1}\alpha_{2}}{d^{6}}=0$$

where $\alpha_{1}$ is the polarizability of the particle at point $r_{1}$ and $\alpha_{2}$ is the polarizability of the particle at point $r_{2}$. The polarizability of a spherical particle in a medium with dielectric constant $\varepsilon_{m}$ is,

(6) $$\alpha_{i}={r_{i}}^{3}\frac{\varepsilon_{i}-\varepsilon_{m}}{\varepsilon_{i}+2\varepsilon_{m}}$$

where $r_{i}$ and $\varepsilon_{i}$ are the radius and the dielectric function of the nanosphere, respectively. Equation (6) can be substituted into Equation (5) for $\alpha_{1}$ and $\alpha_{2}$ to obtain the dispersion equation for two equal coupled spheres.

(7) $$4{\left(\frac{r}{d}\right)}^{6}{\left(\frac{\tilde{\varepsilon }\left(\lambda \right)-\varepsilon_{m}}{\tilde{\varepsilon }\left(\lambda \right)+2\varepsilon_{m}}\right)}^{2}=1$$

By solving Equation (7) with respect to $\tilde{\varepsilon }\left(\lambda \right)$, we find the dielectric function of the two-particle ”cluster” at resonance for antisymmetric plasmon oscillations,

(8) $$\tilde{\varepsilon }\left(\lambda \right)=-2\varepsilon_{m}\left(1+3{\left(\frac{r}{d}\right)}^{3}\right)$$

**Figure 3:**
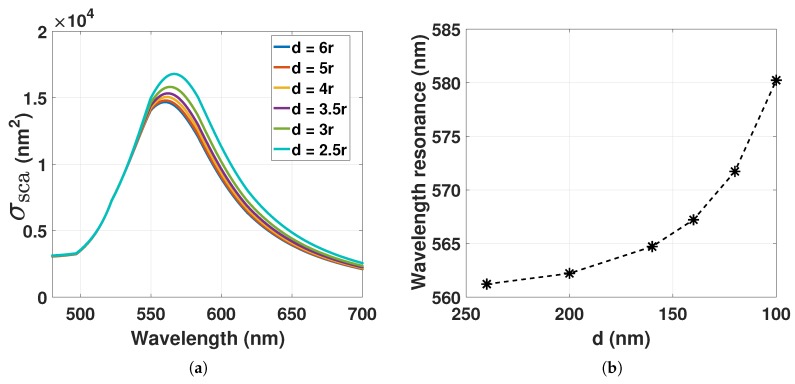
(**a**) Scattering cross-section computed from Equation (1) with L=1 of GNP with 80 nm in diameter for the decrease in *d*; (**b**) LSPR peak position from Figure (**a**) as a function of decreasing *d*. $n_{m}=1.33$.

The resonance condition Reε˜(λ)=−2εm used for describing Equation (4) can then be corrected with Equation (8) to include the dipole-dipole interactions. With $n_{md}=\sqrt{\varepsilon_{m}\varepsilon_{d}}=n_{\mathrm{gel}}\sqrt{1+3{\left(\frac{r}{d}\right)}^{3}}$, where $n_{\mathrm{gel}}=\sqrt{\varepsilon_{m}}$ is the RI of the hydrogel and $\varepsilon_{d}=\left(1+3{\left(\frac{r}{d}\right)}^{3}\right)$, the scattering cross-section of GNP can be computed from Equation (1) with L=1 for decreasing *d* as shown in Figure 3. A decrease in *d* redshifts the LSPR wavelength. With constant $n_{\mathrm{gel}}$ and increasing ND due to hydrogel contraction, the redshift is proportional to ${\left(\frac{r}{d}\right)}^{3}$. The redshift of the decreasing *d* will also be associated with a spectral broadening and an increasing amplitude.

### 2.3. The Influence of the Nearest-Neighbor Distribution Function on the LSPR Signal

The linewidth and the amplitude of the LSPR signal from the GNP-hydrogel depends on the RI of the medium, the radiative and non-radiative damping of the oscillating electrons, as well as the polydispersity index (PDI) of the GNPs ($\mathrm{PDI}=\frac{\mathrm{Variance}}{{\mathrm{Average}}^{2}}$ of the cumulant analysis of the dynamic light scattering of colloids [13]). For hydrogel deswelling, the interparticle distance decreases and increases the dipole-dipole interactions between the GNPs. The resonance condition can be expressed as Equation (8). The linewidth and the amplitude of the LSPR signal depend now also on the distribution of the distances between the particles. The probability density function related to interparticle distances can be accurately estimated by utilizing models based on the nearest-neighbor distribution function (NNDF). Work from Torquato, Lu and Rubinstein has derived theoretical expressions of ‘void’ NNDF of random distributed impenetrable spheres where the probability of finding the nearest neighbor is at a given distance l=d−r from a point in the region exterior to the particles [14,15,16]. The void NNDF for randomly-distributed three-dimensional impenetrable spheres in the Carnahan–Starling approximation can be expressed as,

(9) $$H_{v}\left(y\right)=24\eta \left(1-\eta \right)\left(ey^{2}+fy+g\right)\exp\left[-\eta \left(8ey^{3}+12fy^{2}+24gy+h\right)\right],y>\frac{1}{2}$$

where $y=\frac{l}{2r}$, $\eta =\frac{\varphi \pi {\left(2r\right)}^{3}}{6}$ is the reduced density, φ is the ND and e=e(η), f=f(η), g=g(η), h=h(η) are the density-dependent coefficients.

(10a) $$e\left(\eta \right)=\frac{1+\eta }{{\left(1-\eta \right)}^{3}}$$

(10b) $$f\left(\eta \right)=-\frac{\eta (3+\eta )}{2{\left(1-\eta \right)}^{3}}$$

(10c) $$g\left(\eta \right)=\frac{\eta^{2}}{2{\left(1-\eta \right)}^{3}}$$

(10d) $$h\left(\eta \right)=\frac{-9\eta^{2}+7\eta -2}{2{\left(1-\eta \right)}^{3}}$$

For our experiments, the GNPs have a diameter of 80 nm with ND at $\approx 10^{11}$ particles/mL. Thus, the reduced density for GNPs embedded in the hydrogel becomes $\eta =2.6\times 10^{-5}$. Since η<<1 for the GNP densities used in our experiments, Equation (9) can be simplified to,

(11) $$H_{v}\left(y\right)=24\eta y^{2}\exp\left[-8\eta y^{3}\right],y>\frac{1}{2}$$

Considering that there is significant dipole-dipole interactions between GNPs for d≤5r, the probability of finding particles within this range can be computed by integrating $H_{v}\left(y\right)$ from $y_{1}=\frac{2r-r}{2r}=\frac{1}{2}$ to $y_{2}=\frac{5r-r}{2r}=2$,

(12) $$P\left(d\leq 5r\right)=\int_{y_{1}}^{y_{2}}24\eta y^{2}\exp\left[-8\eta y^{3}\right]dy=-\exp\left[-8\eta y_{2}^{3}\right]+\exp\left[-8\eta y_{1}^{3}\right]$$

For the increase of ND in Equation (11), the probability of finding the nearest-neighbor at a given distance l=d−r is increasing while the width of the distribution function $H_{v}\left(y\right)$ is decreasing. The change in ND for hydrogel swelling or contraction will then change the probability value $H_{v}\left(\frac{\bar{d}-r}{2r}\right)$ at mean interparticle distance ($\bar{d}$) and influence the amplitude and the linewidth of the LSPR signal for $\bar{d}\leq 5r$.

### 2.4. Assessing the Refractive Index for Hydrogel Swelling Degree

We can express the RI of the hydrogel as,

(13) $$n_{\mathrm{gel}}=n_{p}x_{p}+n_{w}\left(1-x_{p}\right)$$

where $n_{p}$ is the RI of the polymer, $n_{w}$ is the RI of the water and $x_{p}$ is the mole fraction of the polymer. As the hydrogel is composed of two components, polymer and water, the relation between mole fraction *x* and mass fraction *w* can be found from:

(14a) $$w_{w}m_{p}=w_{p}m_{w}$$

(14b) $$x_{p}\frac{m_{w}}{M_{w}}=x_{w}\frac{m_{p}}{M_{p}}$$

where $w_{p}$ is the mass fraction of the polymer, $w_{w}$ is the mass fraction of water, $m_{p}$ is the mass of the polymer, $m_{w}$ is the mass of water, $M_{p}$ is the molar mass of the polymer and $M_{w}$ is the molar mass of water. $m_{p}$ will be constant for hydrogel swelling and deswelling, while $m_{w}$ will change. By using the relations in Equation (14a,b) the ratio $\frac{m_{w}}{m_{p}}$ can be expressed with respect to the mole fraction of the polymer as,

(15) $$\frac{m_{w}}{m_{p}}=\left(\frac{1}{x_{p}}\right)\left(\frac{M_{w}}{M_{p}+M_{w}\frac{w_{p}}{w_{w}}}\right)=\frac{1}{x_{p}}a$$

$x_{p}$ can be further described as a function of the hydrogel swelling degree by scaling it to the ratio $\frac{V_{\mathrm{pregel}}}{V}$ where $V_{\mathrm{pregel}}$ is the volume of pregel on the OF and *V* is the volume of the hydrogel on the OF.

The ratio $\frac{m_{w}}{m_{p}}$ as a function of $\frac{V_{\mathrm{pregel}}}{V}=v$ by the use of Equation (14a) becomes,

(16) $$\frac{m_{w}}{m_{p}}=\left(\frac{1}{v}\right)\left(\frac{\frac{w_{w0}}{w_{p0}}\rho_{p}+\rho_{w}}{\rho_{p}+\frac{w_{p}}{w_{w}}\rho_{w}}\right)=\frac{1}{v}b$$

where $\rho_{p}$ is the density of the polymer (pure) and $\rho_{w}$ is the density of water (pure). By setting Equation (15) equal to (16), $x_{p}$ is scaled to *v* with *a* and *b*, 

(17) $$x_{p}=v\frac{a}{b}$$

The ratio $\frac{a}{b}$ is only weakly dependent on the swelling degree. We can therefore approximate $\frac{a}{b}$ as a constant found with the initial value of $x_{p0}$ and $v_{0}$. By inserting Equation (17) into Equation (13), the RI can be assessed for the hydrogel for swelling and contraction.

## 3. Materials and Methods

### 3.1. Materials

The gels were prepared by using the following chemicals: acrylamide (AAM) (99%, Sigma Aldrich, Schnelldorf, Germany), acrylic acid (AAC) (99%, Sigma Aldrich), *N*,*N*-methylenebisacrylamide (BIS) (≥99.5%, Sigma Aldrich), 1-hydroxycyclohexyl phenyl ketone (99%, Sigma Aldrich), dimethyl sulfoxide (DMSO) (≥99.9%, Sigma Aldrich), octamethylcyclotetrasiloxane (98%, Sigma Aldrich), 3-(trimethoxysilyl) propyl methacrylate (Silane A174) (98%, Sigma Aldrich), citrate-stabilized spheroidal GNPs of 80 nm in diameter ($7.8\times 10^{9}$ particles/mL, absorption max: 551–557 nm, PDI≤0.2, Sigma Aldrich), phosphate-buffered saline (PBS) (Tablet, Sigma Aldrich) and squalane (99%, Sigma Aldrich). Milli-Q (mQ) water (resistivity 18.2 M/cm, Millipore Simplicity 185) was used for all solutions. Hydrochloric acid (HCl) (1.0 M, Sigma Aldrich) and sodium chloride (NaCl) (18% *w*/*v*, VWR, Oslo, Norway) were added to mQ-water to prepare solutions for controlling the hydrogel swelling and contraction. The GNP solution was densified to an ND of $1.95\times 10^{11}$ particles/mL by water evaporation. AAM, AAC and BIS were dissolved in PBS solution (pH 7.4) to prepare a stock solution with 30 wt% AAM-AAC with a molar ratio of 15/85 AAM/AAC and with 2 mol% BIS. A pregel solution of 10 wt% AAM-AAC and 2 mol% BIS was prepared by adding citrate-stabilized GNP or PBS to the AAM-AAC stock solution of 30 wt%.

### 3.2. Synthesize Hydrogel on OF End Face

The LSPR OF segment in Figure 1 was based on Ø200μm MM OF (FT200EMT, Thorlabs, Göteborg, Sweden) that were stripped of the jacket and cleaned with 96% ethanol, cut (Cleaver MS-7310, Melbye Skandinavia, Oslo, Norway) and prepared for silanization [17]. For silanization of the OF end face, the tips of the OF were soaked in a solution of 0.01M HCl for 15 min to activate the surface, cleaned with mQ-water and then immersed in a solution of 3-(trimethoxysilyl) propyl methacrylate (0.084 M, nitrogen purged octamethylcyclotetrasiloxane) for 10 min. The OF was then cleaned with 96% ethanol and stored for up to two weeks. The pregel solutions from Section 3.1 were used further for the synthesis of hydrogel on the silanized OF end face. Then, 0.01 M 1-hydroxycyclohexyl phenyl ketone photoinitiator (PI) in DMSO was added to the pregel solution to a volume ratio of 31/2000 PI/pregel, so a final pregel solution was made. A drop of squalane added with PI (2.7 mg/mL) was deposited on a glass rod. The silanized OF was located in the squalane-PI drop, and an aliquot of the final pregel solution was transferred to its end face by a pipette (Finnpipette F2, Thermo Scientific, Oslo, Norway). Next, the gel-OF was aligned with an ultraviolet (UV) Ø365μm Core MM OF (FG365UEC, Thorlabs, Göteborg, Sweden) by the use of an optical stage under observation in an optical stereo-microscope (SZX7, Olympus, Oslo, Norway). The UV-OF illuminated the gel-OF with light at 365 nm by the use of a fiber-coupled LED (M365F1, Thorlabs, Göteborg, Sweden), and it was cured for 10 min. The polymerized gel-OF was subsequently immersed in pentane to remove impurities for 5 s and transferred to PBS solution until further use.

### 3.3. Setup of the Fiber Optic Instrument

**Figure 4:**
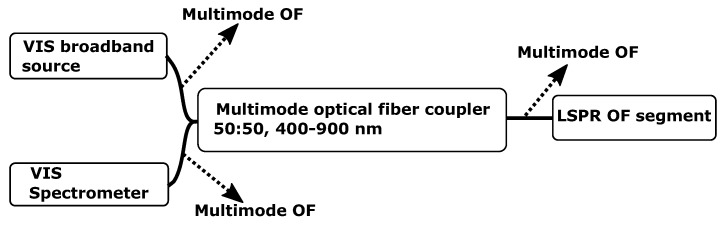
Configuration of the reflection-based FO system containing the light source, spectrometer, OF coupler and LSPR OF segment.

The FO setup illustrated in Figure 4 consists of the following components: visible (VIS) broadband source (HL-2000-FHSA-LL, 360–2400 nm, Ocean Optics, Oslo, Norway), 50:50 coupler MM (50/50, FCMH2-FC, 400–1600 nm, Thorlabs, Göteborg, Sweden), VIS spectrometer (QE65Pro, Ocean Optics, Oslo, Norway), loose OF-ends terminated with index matching gel (G608N3, Thorlabs, Göteborg, Sweden), LSPR OF segment Ø200μm MM OF (FT200EMT, Thorlabs, Göteborg, Sweden).

The data acquisition was obtained with the program Spectrasuite (Ocean Optics, Oslo, Norway), and the OFs were spliced using a Fitel Fusion Splicer (Furukawa Electric, Tokyo, Japan).

### 3.4. Reflectance Measurements of GNP Embedded in Hydrogel

The reflectance spectra were estimated from the measured raw spectra $S_{\lambda }$ normalized to a measured reference spectrum $R_{\lambda }$. Before normalization, we subtracted the measured dark spectrum $D_{\lambda }$ (recorded with the light source turned off) from both the raw spectra and reference spectra. The normalized reflectance spectra were then computed as:

(18) $$I_{R}=\left(\frac{S_{\lambda }-D_{\lambda }}{R_{\lambda }-D_{\lambda }}\right)\times 100\%$$

The reference spectrum was recorded from the reflections of the bare Ø200μm MM OF in mQ-water solution. To determine the LSPR peak position, the reflectance spectra were fitted with a centered and scaled smoothing spline function with the smoothing parameter at 0.999. With smoothing parameter p=0, the smoothing spline function produces a least-square line fit to the data, whereas with p=1, the smoothing spline function produces a cubic spline interpolant. By choosing a fixed smoothing parameter, the balance between residual error and local variation is also fixed [18].

Scattering increases with increasing GNP size, with an associated spectral broadening of the LSPR signal. Thus, for our FO system with GNP diameter ≥80 nm and density $\geq 2\times 10^{10}$ particles/mL in mQ-water solution, we observed a high LSPR signal. The hydrogels used in our swelling measurements were polymerized from pregels with 80-nm GNP densities at ${\mathrm{ND}}_{01}=8.86\times 10^{9}$ particles/mL, ${\mathrm{ND}}_{02}=1.73\times 10^{10}$ particles/mL and ${\mathrm{ND}}_{03}=1.7\times 10^{11}$ particles/mL.

The hydrogel contraction and swelling were controlled by immersing the gel-OF in pH solutions between 5 and 3 with a constant ionic strength (IS) at 0.274 M. The reflectance spectra were recorded after contraction or swelling had reached equilibrium. The gel-OF was washed in PBS after each measurement to control the size of hydrogel for deswelling only. pH and IS were controlled with a pH/IS meter (inoLab pH/ION 7320, WTW, Oslo, Norway), electrode selective towards Cl (Cl 800 (BNC), WTW, Oslo, Norway), pH electrode (pHenomenal MIC 220, VWR Collection, Oslo, Norway) and temperature measurer (pHenomenal TEMP21, VWR Collection, Oslo, Norway). All the experiments were carried out at room temperature, and the pH and IS of the solutions were controlled by adding HCl and NaCl to mQ-water.

## 4. Results

First, the NNDF was estimated for increasing GNP densities in hydrogel to assess the distribution of interparticle distances for the hydrogel swelling degree. The RI as a function of hydrogel deswelling was estimated based on the estimation of the mole fraction of polymer and water. Second, the sources of reflections in the hydrogel were investigated with respect to the LSPR reflectance spectrum to determine possible errors included in the value of the LSPR peak with corresponding wavelength. Third, the LSPR response was demonstrated by measuring its peak positions as a function of GNP-hydrogel contraction controlled with pH solutions and as a function of increasing ${\mathrm{ND}}_{0}$. Last, the linewidth and the amplitude of the LSPR signal were characterized for hydrogel deswelling stimulated with pH solutions to compare the LSPR reflectance spectrum with the estimated NNDF of GNPs in hydrogel.

### 4.1. Nearest-Neighbor Distribution Function for Increasing GNP Density in the Hydrogel

**Figure 5:**
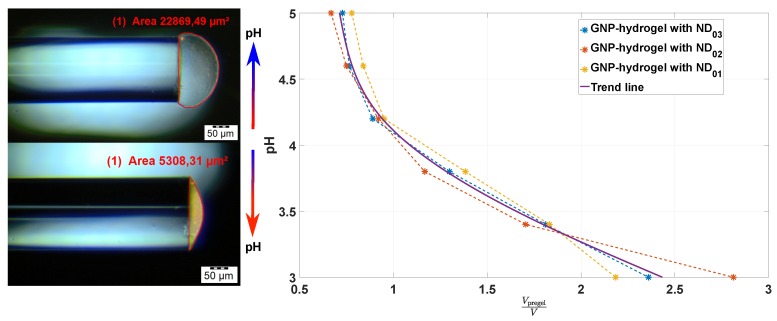
Estimate of the hydrogel volume for the decrease in pH from 5 to 3 based on observations in an optical microscope. The uncertainties of the volume measurements are estimated to be within 1 and 5%. ND, number density.

Figure 5 shows the measured volume of pregel to the volume of hydrogel ($\frac{V_{\mathrm{pregel}}}{V}$) ratio based on the observations obtained from optical microscope imaging.

Three different pregels with different GNP densities at ${\mathrm{ND}}_{01}=8.86\times 10^{9}$, ${\mathrm{ND}}_{02}=1.73\times 10^{10}$ and ${\mathrm{ND}}_{03}=1.7\times 10^{11}$ particles/mL were used to manufacture the OF sensors. The ratio $\frac{V_{\mathrm{pregel}}}{V}$ was estimated for hydrogel contraction controlled with pH solutions from 5 to 3. $\frac{V_{\mathrm{pregel}}}{V}$ as a function of decreasing pH follows the same trend for the OF sensors fabricated from different densities of GNP in pregel. The NDs of GNPs found for the deswelling hydrogels are further used to compute the NNDF.

**Figure 6:**
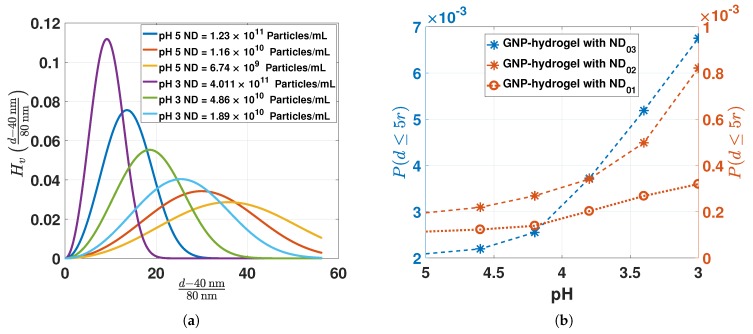
(**a**) Computation of the void nearest-neighbor distribution function (NNDF) (Equation (11)) for the NDs estimated in Figure 5 for pH 5 and 3; (**b**) computed probability (Equation (12)) for the interparticle distances between 2r≤d≤5r from the ND estimated in Figure 5.

In Figure 6a, the NNDFs (Equation (11)) are computed for pH 5 and 3 (from the NDs in Figure 5) of the OF sensors manufactured from different GNP-pregel densities. The probability at the mean interparticle distance $\bar{d}$ is increasing for increasing ND and hydrogel contraction. The smallest $\bar{d}$ for the largest ND and lowest pH is greater than 5r, i.e., greater than the range where the interaction between dipoles of GNPs occurs.

The probability (Equation (12)) of finding particles with interparticle distance between 2r≤d≤5r (computed from NDs in Figure 5) is represented in Figure 6b. The probability is increasing from $0.1\times 10^{-3}$ to $6.7\times 10^{-3}$ for increasing ND and hydrogel contraction. The low probability for 2r≤d≤5r shows that there is only a small fraction of GNPs with interparticle distances inducing dipole-dipole interactions. Hence, for a truly random distribution of GNPs in hydrogel at pH 5 and 3, it is the increasing density of the polymer network and not the dipole-dipole interactions that should be the dominating factor for the change in the LSPR peak position. If the change in LSPR peak position is independent of ND in our experiments, the assumption of having particles randomly distributed in the hydrogel would be reasonable. On the contrary, if the LSPR peak position is dependent on the ND, the GNPs may be inhomogeneously distributed in the hydrogel with a large fraction of particles with d≤5r.

### 4.2. Refractive Index as a Function of Hydrogel Swelling Degree

**Figure 7:**
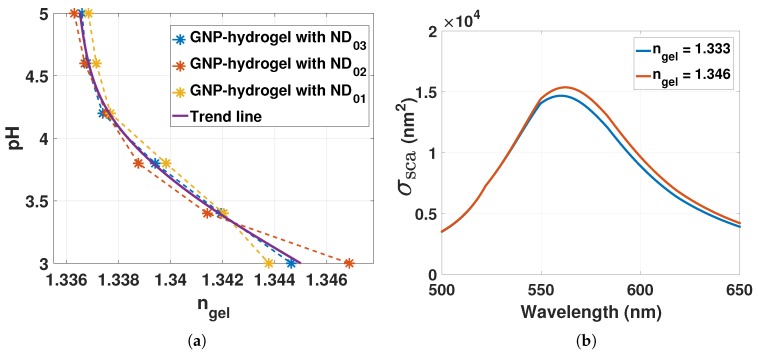
(**a**) RI of hydrogel computed from Equation (13) and (17) for pH 5 to 3; (**b**) scattering cross-section computed from Equation (1) with L=1 for $n_{m}=n_{\mathrm{gel}}$ at 1.333 and 1.346.

The RI of the hydrogel can be assessed from Equation (13) by computing the mole fraction of the polymer in Equation (17). The RI of the polymer can be assumed to be dominated by the mole and mass fraction of AAM-AAC. For our experiments, the mass fraction of AAM-AAC is $w_{p0}=0.1$. With $n_{p}=1.513$ and $n_{w}=1.333$ [19,20], $n_{\mathrm{gel}}$ from Equation (13) and (17) can be computed as a function of $\frac{V_{\mathrm{pregel}}}{V}$ (from Figure 5) for pH 5 to 3 as presented in Figure 7a.

Deswelling of the hydrogel for pH 5 to 3 increases $n_{\mathrm{gel}}$ from ∼1.333 to ∼1.346. Inserting $n_{m}=n_{\mathrm{gel}}$ at 1.333 and 1.346 into Equation (1) with L=1, the scattering of GNP can be computed as shown in Figure 7b.

The scattering of GNPs redshifts by 2 to 3 nm with hydrogel contraction stimulated with pH from 5 to 3. Its important to note that the LSPR is dependent on the mole fraction of polymer chains that are in close proximity to the plasmonic wave of the GNPs. Due to the localized sensing of the RI changes on the surface of the GNPs, $n_{m}$ in Equation (1) and $n_{\mathrm{gel}}$ in Equation (13) would rather represent the local RI and the bulk RI of the hydrogel, respectively. Hence, the LSPR response of GNPs could be different for bulk and local RI changes.

### 4.3. The Reflectance for Different GNP Densities

**Figure 8:**
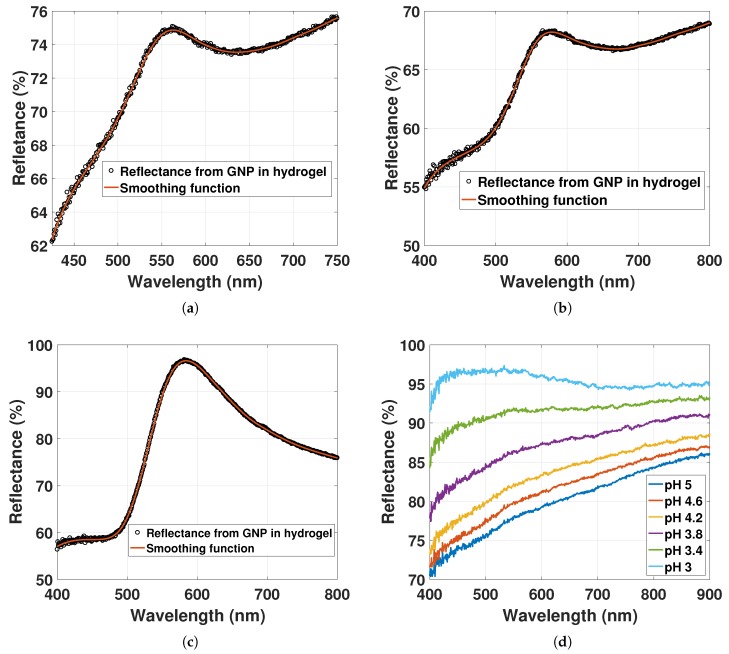
(**a**) Reflectance of GNP immobilized in hydrogel with ND of $6.74\times 10^{9}$ particles/mL and (**b**) $1.6\times 10^{10}$ particles/mL in solution of pH at 5; (**c**) reflectance of GNP immobilized in hydrogel with ND of $1.36\times 10^{11}$ particles/mL in solution of pH at 4.4; (**d**) reflectance from hydrogel without GNPs for pH 5 to 3.

Figure 8a–c shows reflectance spectra for $6.74\times 10^{9}$ particles/mL (pH 5), $1.6\times 10^{10}$ particles/mL (pH 5) and for $1.36\times 10^{11}$ particles/mL (pH 4.4), respectively (NDs found from Figure 5). The reflectance measurements of hydrogel without GNPs are shown in Figure 8d for pH 5 to 3.

The LSPR peaks are at 561 nm, 575 nm and 583 nm for $6.74\times 10^{9}$ particles/mL, $1.6\times 10^{10}$ particles/mL and $1.36\times 10^{11}$ particles/mL, respectively. The increasing ND of GNPs is redshifting the LSPR signal, while its linewidth is broadening. That the LSPR peak position is dependent on the ND indicates an electrostatic interaction between the dipoles. This contrasts with the computed NNDF in Section 4.1, where only a minor fraction of GNPs was estimated to have interparticle distances less than 5r. The particles in the hydrogel may not exhibit a random distribution, but could rather exhibit an inhomogeneous distribution with a large fraction of GNPs with interparticle distances between 2r≤d≤5r.

**Figure 9:**
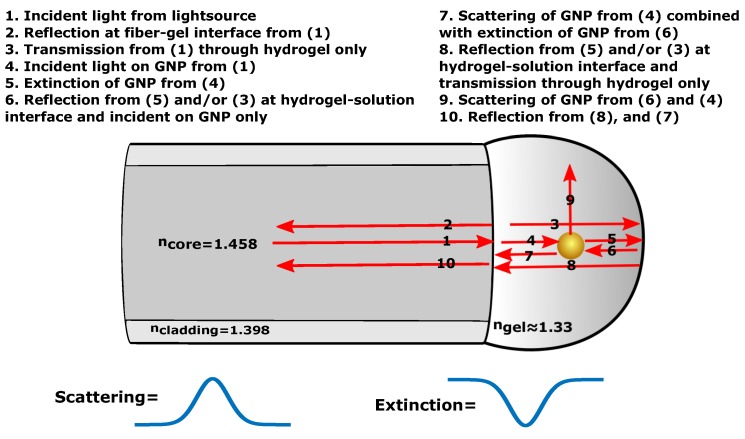
Sources of scattering and extinction from hydrogel and GNP.

The spectra in Figure 8a–c may contain reflections other than scattering from GNP that could result in errors in the LSPR peak position value. Not only light scattering, but also light extinction of GNP occurs due to the reflection at the hydrogel-solution interface. The extinction of GNPs will add a spectrum of opposite sign to the LSPR signal relative to the scattered spectrum of GNP. The sources of scattering and extinction of GNP and hydrogel can be listed as illustrated in Figure 9.

The reflection at a normal incidence (2) can be assumed to be roughly 0.002 at the fiber-gel interface. The reflection (8, 6) at the hydrogel-solution interface has in previous work been determined based on visibility measurements [21]. With reflection at 0.002 for the fiber-gel interface and with visibility at 0.2, the reflection (8, 6) at the hydrogel-solution interface can be estimated to be 0.0002. Multiple reflections between the hydrogel-solution and fiber-gel interface can then be safely neglected. Hence, at the LSPR wavelength, the scattering combined with extinction of GNP (7) is dominated by the scattering from (4) as its intensity is much higher than the reflection from (6).

The reflectance measurements of hydrogel without GNP in Figure 8d have a decreasing slope and an increasing reflectance from pH 5 to 3. The change in the slope from the reflectance of the hydrogel will also change the observed LSPR peak position from the reflectance of the GNPs. The reflectance of GNP-hydrogel can be modeled with two functions, the reflectance of the hydrogel without GNPs and the LSPR signal from the GNPs, here modeled as a Gaussian function $g(\lambda -\lambda_{0})$. Thus, we can write,

(19a) $$f_{1}\left(\lambda \right)=g\left(\lambda -\lambda_{0}\right)+v_{0}f_{2}\left(\lambda \right)$$

(19b) $$f_{2}\left(\lambda \right)=a_{2}{\left(\lambda -\lambda_{0}\right)}^{2}+a_{1}\left(\lambda -\lambda_{0}\right)+a_{0}$$

where $f_{1}\left(\lambda \right)$ represents the reflection of both GNP and hydrogel with $v_{0}$ as a scaling factor for the reflection from the hydrogel without GNPs, $f_{2}\left(\lambda \right)$.

Assuming that $a_{2}<<a_{1}$, the LSPR peak position as a function of $f_{1}\left(\lambda \right)$ can be described by setting $\frac{\partial f_{1}\left(\lambda \right)}{\partial \lambda }=0$,

(20) $$\lambda_{\max}=\frac{v_{0}a_{1}\sigma^{2}}{b}+\mu$$

where $\sigma =\frac{\mathrm{FWHM}}{2\sqrt{\ln2}}$. μ and g(μ)=b are the peak position and amplitude, respectively, of the LSPR signal represented as $g(\lambda -\lambda_{0})$. The derivative of $\lambda_{\max}$ with respect to $a_{1}$ determines the shift of $\lambda_{\max}$ with changing slope $a_{1}$,

(21) $$\Delta \lambda_{\max}=\frac{v_{0}\sigma^{2}}{b}\Delta a_{1}$$

For the reflectance of hydrogel without GNPs in Figure 8d, $\Delta a_{1}$ can be estimated to be $-0.008\frac{\%}{\mathrm{nm}}$ with $a_{2}<<a_{1}$ for the change in pH from 5 to 3.8. The factor $\frac{v_{0}\sigma^{2}}{b}=\bar{m}$ from the LSPR signal will also determine the shift $\Delta \lambda_{\max}$. For the GNP-hydrogel with low ND in Figure 8a,b, $\bar{m}$ will be large. Thus, an increase in the slope will lead to a redshift of the LSPR signal, whereas a decrease in slope will lead to a blueshift. For the GNP-hydrogel with high ND in Figure 8c, $\bar{m}$ will be small. The increase or decrease in slope will in this case lead to negligible red or blueshifts of the LSPR signal.

### 4.4. LSPR Peak Position as a Function of Hydrogel Swelling Degree

**Figure 10:**
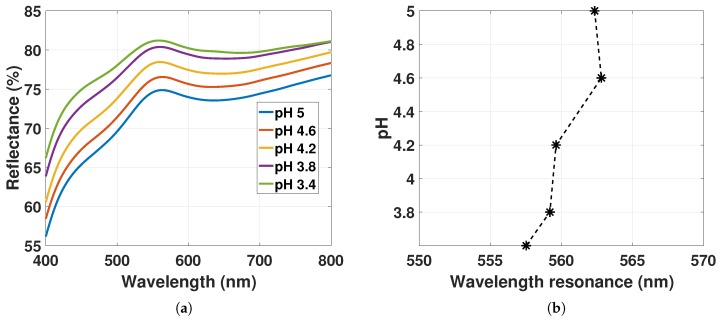
(**a**) Reflectance spectra of GNP-hydrogel with ${\mathrm{ND}}_{01}$ at $8.86\times 10^{9}$ particles/mL fitted with a smoothing spline function for pH 5 to 3.4; (**b**) LSPR peak position from Figure 10a as a function of hydrogel deswelling stimulated with pH solutions from 5 to 3.4.

The reflectance spectra of GNP-hydrogel with ${\mathrm{ND}}_{01}$ at $8.86\times 10^{9}$ particles/mL fitted with a smoothing spline function are shown in Figure 10a for pH solutions from 5 to 3.4. The resulting LSPR peak positions as a function of hydrogel contraction stimulated with pH solutions are presented in Figure 10b.

The reflectance peak is increasing for decreasing pH. As discussed in Section 4.3 for the OF sensor with ${\mathrm{ND}}_{01}$, the reflectance spectrum will be a sum of the scattering from GNPs with an added slope from the light extinction of the hydrogel. The increase in reflectance peak in Figure 10a may be due to the increase in the reflectance of the hydrogel contraction as observed from Figure 8d. The LSPR peak positions in Figure 10b are changing from 562 nm to 557 nm as a function of hydrogel deswelling. For decreasing pH, the slope from the reflectance of the hydrogel is declining as discussed in Section 4.3. From pH 5 to 3.8, the linear function $f_{2}\left(\lambda \right)$ in Figure 10a has a change in slope of $\Delta a_{1}=-0.01\frac{\%}{\mathrm{nm}}$ between 450 nm and 750 nm that is comparable to $\Delta a_{1}=-0.008\frac{\%}{\mathrm{nm}}$ for the reflectance of hydrogel without GNPs in Figure 8d. $\Delta \lambda_{\max}$ from Equation (21) can be estimated to be ∼2.25 nm based on the reflectance of GNP-hydrogel in Figure 10a (setting $\Delta a_{1}=-0.01\frac{\%}{\mathrm{nm}}$, FWHM = 61 nm, $g(\lambda -\lambda_{0})=3\%$ and $v_{0}=1$). As discussed in Section 4.2, an increasing mole fraction of polymer in close proximity to the plasmonic wave of the GNPs could also redshift the LSPR signal by 2 to 3 nm. Hence, the blueshift of the LSPR peak in Figure 10b is due to the reduced slope from the reflectance of hydrogel for pH 5 to 3.4, as well as a result of the local variations of the RI in the hydrogel for the increasing mole fraction of polymer. With ${\mathrm{ND}}_{01}$ at $8.86\times 10^{9}$ particles/mL, there is then little contribution from the dipole-dipole interactions on the LSPR signal.

**Figure 11:**
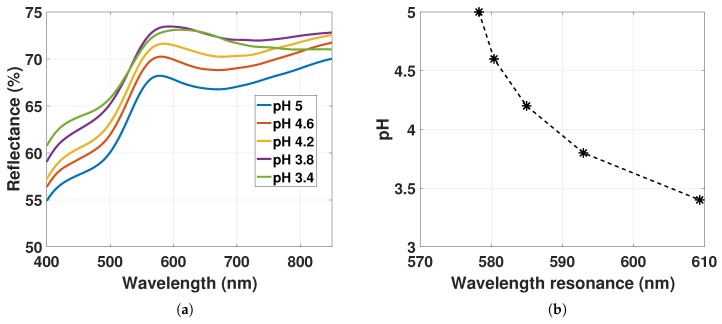
(**a**) Reflectance spectra of GNP-hydrogel with ${\mathrm{ND}}_{02}$ at $1.73\times 10^{10}$ particles/mL fitted with a smoothing spline function for pH 5 to 3.4; (**b**) LSPR peak position from Figure 11a as a function of hydrogel deswelling stimulated with pH solutions from 5 to 3.4.

Figure 11 represents the reflectance spectra of GNP-hydrogel with increased ND to ${\mathrm{ND}}_{02}$ at $1.73\times 10^{10}$ particles/mL fitted with a smoothing spline function for hydrogel deswelling and the corresponding LSPR peak positions.

The reflectance peak is increasing for pH 5 to 3.8. The increase may be a result of the increase in reflectance of the hydrogel for declining pH as discussed in Section 4.3. For pH 3.8 to 3.4, the ND of GNPs in the hydrogel increases from $2.02\times 10^{10}$ particles/mL to $2.95\times 10^{10}$ particles/mL (estimated from data in Figure 5), whereas the amplitude is declining and the linewidth is broadening (linewidth and amplitude estimated in Section 4.5). The decline in the reflectance peak from pH 3.8 to 3.4 is due to the decrease in the amplitude of the LSPR signal that is smaller than the increase in the reflectance of the hydrogel observed in Figure 8d. A decreasing amplitude for higher ND suggests also the hypothesis that there is a larger dispersion in particle scattering, which may be caused by the increased fraction of particles interacting, as well as the increased variation in local RI. The linewidth broadening is likely due to the increasing RI and the decrease in $\bar{d}$ inducing dipole-dipole interactions.

The LSPR peak positions in Figure 11a as a function of hydrogel contraction controlled with pH solutions are presented in Figure 11b. The LSPR peak position is redshifting from 578 nm to 610 nm from pH 5 to 3.4. The change in the LSPR peak position in Figure 11b for decreasing pH is now due to three factors: (1) the RI change as a function of hydrogel contraction presented in Section 4.2 results in a redshift of 2 to 3 nm; (2) the change in the slope of $\Delta a_{1}=-0.015\frac{\%}{\mathrm{nm}}$ from pH 5 to 3.8 in Figure 11a that results in a blueshift of 2 to 2.5 nm (see discussion from Figure 10); (3) the decrease in $\bar{d}$ inducing electrostatic interactions between of the GNPs results in redshift of the LSPR signal. The change in LSPR peaks in Figure 10 and Figure 11 shows a different response to the hydrogel deswelling due to the different amounts of GNPs immobilized in the hydrogel. By changing the density of GNPs in pregel from ${\mathrm{ND}}_{01}$ to ${\mathrm{ND}}_{02}$, the LSPR response for hydrogel contraction becomes dependent on the decreasing $\bar{d}$ as a result of the increasing fraction of GNPs with $\bar{d}\leq 5r$. Whereas the results in Figure 10 show a small blueshift of the LSPR due to the change in the slope $\Delta a_{1}$ with little influence from the dipole-dipole interactions, the results in Figure 11 show a large redshifting of the LSPR due to the large influence of dipole-dipole interactions and with little influence from the slope $\Delta a_{1}$.

**Figure 12:**
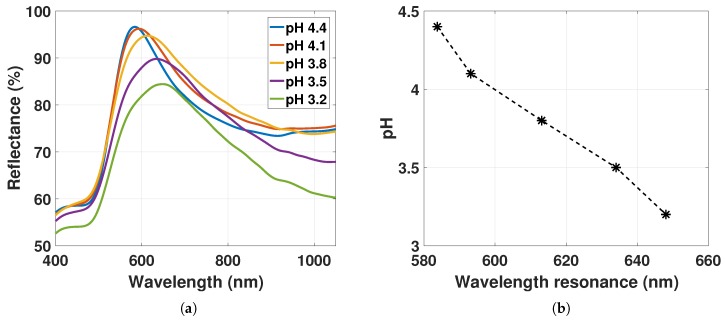
(**a**) Reflectance spectra of GNP-hydrogel with ${\mathrm{ND}}_{03}$ at $1.7\times 10^{11}$ particles/mL fitted with a smoothing spline function for pH 4.4 to 3.2; (**b**) LSPR peak position from Figure 12a as a function of hydrogel deswelling stimulated with pH solutions from 4.4 to 3.2.

Finally, the LSPR peak position was measured as a function of hydrogel deswelling stimulated with decreasing pH with even higher ${\mathrm{ND}}_{03}$ at $1.7\times 10^{11}$ particles/mL in Figure 12.

The reflectance spectra of GNP-hydrogel for pH 4.4 to 3.2 fitted with a smoothing spline function are shown in Figure 12a, while the LSPR peak positions as a function of decreasing pH are presented in Figure 12b. The reflectance peak is decreasing, while the linewidth of the reflectance is broadening for hydrogel contraction. From pH 4.4 to 3.2, the ND increases from $1.4\times 10^{11}$ particles/mL to $3.5\times 10^{11}$ particles/mL (estimated from Figure 5). It is evident that the change in the amplitude of the LSPR signal from scattering of GNPs in Figure 12a dominates over the change in reflectance of the hydrogel observed in Figure 8d for the decrease in pH (linewidth and amplitude estimated in Section 4.5).

The decrease in amplitude of the LSPR signal from pH 4.4 to 3.2 may be a result of the increasing ND of GNPs from $1.4\times 10^{11}$ particles/mL to $3.5\times 10^{11}$ particles/mL (from data in Figure 5) that increases the dispersion in particle scattering caused by the increased fraction of particle interaction and the increased change in the variations of the local RI. The linewidth broadening is likely due to the increase in RI and the decrease in $\bar{d}$ inducing dipole-dipole interactions.

In Figure 12a, the LSPR peak position is redshifting from 584 nm to 648 nm for hydrogel deswelling. The change of the shift is 64 nm, which is much larger than the shift of 2 to 3 nm from the change in RI for hydrogel contraction discussed in Section 4.2. $\Delta \lambda_{\max}$ from Equation (21) can be estimated to be ∼2.65 nm (with $\Delta a_{1}=-0.01\frac{\%}{\mathrm{nm}}$, with $g(\lambda -\lambda_{0})=38\%$ and FWHM = 237 nm). With $\Delta \lambda_{\max}\approx 2.65$ nm and minor RI changes for hydrogel contraction, the redshift of LSPR peak position observed in Figure 12b is most likely dominated by the reduced $\bar{d}$ inducing dipole-dipole interactions between the GNPs.

### 4.5. Amplitude and Linewidth of the LSPR Signal as a Function of Hydrogel Swelling Degree

**Figure 13:**
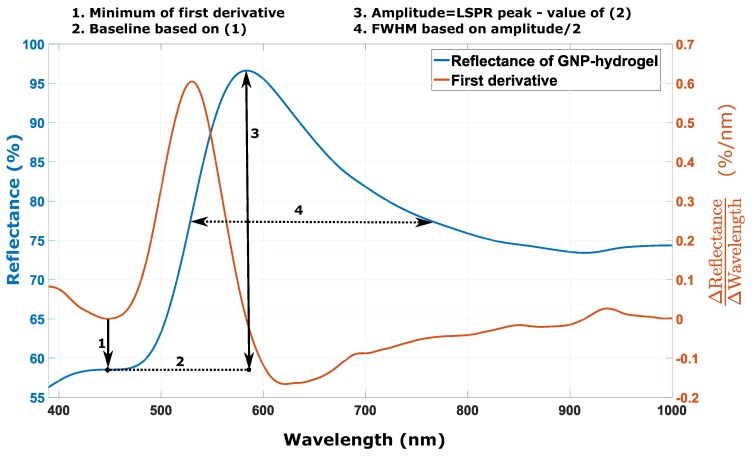
Smoothing fitted function of the reflectance of GNP-hydrogel with ${\mathrm{ND}}_{03}$ at $1.7\times 10^{11}$ particles/mL at pH 4.4 with the corresponding first derivative. The minimum of the first derivative of the reflectance of GNP-hydrogel determines the baseline used for computing the FWHM.

The amplitude and the linewidth of the LSPR signal from the reflectance spectra of GNP-hydrogel with ${\mathrm{ND}}_{03}$ at $1.7\times 10^{11}$ particles/mL for decreasing pH were determined based on the procedure illustrated in Figure 13.

**Figure 14:**
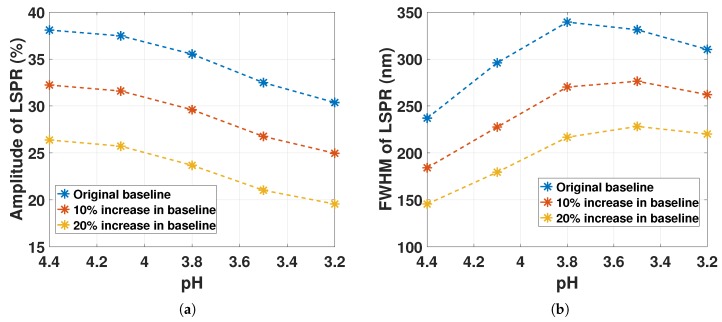
(**a**) Amplitude from the reflectance of GNP-hydrogel with ${\mathrm{ND}}_{03}$ at $1.7\times 10^{11}$ particles/mL for decreasing pH with an increasing baseline; (**b**) FWHM from the reflectance of GNP-hydrogel with ${\mathrm{ND}}_{03}$ at $1.7\times 10^{11}$ particles/mL for decreasing pH with an increasing baseline.

The first inflection point of the LSPR signal is used as the baseline for determining the amplitude and the linewidth of the reflectance of GNP-hydrogel. The LSPR reflectance peak subtracted from the baseline represents the amplitude. The linewidth of the LSPR signal is defined as the FWHM from the half maximum value. Due to the asymmetry of the signal, the procedure will tend to overestimate the linewidth. To account for this, the FWHM was further characterized by varying the baseline with respect to the inflection point. The baseline increasing by 10% and 20% proportionally to the inflection point from Figure 13 reduces both the FWHM and the amplitude, but will represent a value less dependent on the change in the asymmetry of the LSPR signal. Figure 14 shows the resulting amplitude and FWHM from the reflectance spectra of GNP-hydrogel from Figure 12 as a function hydrogel contraction. In Figure 14a, the amplitude of the LSPR signal is decreasing monotonically for pH 4.4 to 3.2, independent of the baseline. The LSPR peak position response in Figure 12 was concluded to be dominated by the reduced $\bar{d}$, inducing dipole-dipole interactions between the GNPs with minor influence from the local variations of the RI change in the hydrogel and the change in the slope $\Delta a_{1}$ from the light extinction of hydrogel for decreasing pH. The declining amplitude in Figure 14a would imply as discussed in Section 4.4 a larger dispersion in particle scattering for higher ND. A larger dispersion in particle scattering may be caused by the increased fraction of interacting particles and the variations in the local RI.

The FWHM in Figure 14b is increasing from pH 4.4 to 3.5. From pH 3.5 to 3.2, the FWHM decreases for the 10% and the 20% increase in the baseline, while for the original baseline, it decreases from pH 3.8 to 3.2. The increase in FWHM is likely a result of the increasing RI and the dipole-dipole interaction between the GNPs. The decrease in FWHM from pH 3.8 to 3.2 might be due to the change in the reflection of the fiber-gel interface observed in Figure 8d, which changes the signature of the LSPR signal.

## 5. Conclusions

A new LSPR-based FO system was developed by immobilizing GNPs in a polymerized polyacrylamid-co-acrylic acid network as a hemispherical hydrogel on OF end face. Proof-of-concept experiments have been presented where we explore the LSPR and interparticle distance distribution attributes of the GNP-hydrogel. The results from the NNDF computation showed that the mean interparticle distance $\bar{d}$ with the GNP densities used in our experiments is much larger than 5r, whereas the results from the reflectance spectra of GNP-hydrogel on OF end face showed that LSPR peak position is dependent on $\bar{d}$. This contradiction suggests that the particles in the hydrogel may not exhibit a random distribution, but could rather exhibit an inhomogeneous distribution with a large fraction of GNPs with interparticle distances between 2r≤d≤5r. The amplitude of the LSPR signal decreases with hydrogel contraction, suggesting the hypothesis that higher ND results in larger dispersion in particle scattering caused by the increased fraction of particles interacting and the increased variations in local RI. The FWHM were increasing from pH 4.4 to 3.5 and decreasing from pH 3.5 to 3.2. The increase in FWHM is likely a result of the increasing RI and the dipole-dipole interactions. The decrease in FWHM from pH 3.8 to 3.2 might be due to the change in the reflection of the fiber-gel interface that changes the signature of the LSPR signal.

Further work will consist of developing the LSPR FO system towards biosensor applications where specific biochemicals will be detected by the use of stimuli-responsive materials embedded with noble metal or silicon nanoparticles (NMDNP). The potential for multi-parametric and label free sensing in complex biological mixtures by the use of an LSPR-based FO biosensor will also be investigated with the focus on the utilization of NMDNP of different sizes, shapes and material compositions. The different NMDNP embedded in tailored polymer networks will then have spectrally-resolvable LSPR peaks where each peak is associated with the detection of a specific biologic entity. By controlling the polarization of the incident light on NMDNP, the sensitivity of LSPR-based FO sensors can be improved by utilizing the dynamic peak position of LSPR for variable light polarizations as the sensing parameter.
